# Evaluation of GO-based functional similarity measures using *S. cerevisiae *protein interaction and expression profile data

**DOI:** 10.1186/1471-2105-9-472

**Published:** 2008-11-06

**Authors:** Tao Xu, LinFang Du, Yan Zhou

**Affiliations:** 1 Shanghai-MOST Key Laboratory of Health and Disease Genomics, Chinese National Human Genome Center at Shanghai, Shanghai 201203, PR China; 2 College of Life Sciences, Sichuan University, Chengdu 610064, PR China; 3 Department of Microbiology, School of Life Sciences, Fudan University, Shanghai 200433, PR China

## Abstract

**Background:**

Researchers interested in analysing the expression patterns of functionally related genes usually hope to improve the accuracy of their results beyond the boundaries of currently available experimental data. Gene ontology (GO) data provides a novel way to measure the functional relationship between gene products. Many approaches have been reported for calculating the similarities between two GO terms, known as semantic similarities. However, biologists are more interested in the relationship between gene products than in the scores linking the GO terms. To highlight the relationships among genes, recent studies have focused on functional similarities.

**Results:**

In this study, we evaluated five functional similarity methods using both protein-protein interaction (PPI) and expression data of *S. cerevisiae*. The receiver operating characteristics (ROC) and correlation coefficient analysis of these methods showed that the maximum method outperformed the other methods. Statistical comparison of multiple- and single-term annotated proteins in biological process ontology indicated that genes with multiple GO terms may be more reliable for separating true positives from noise.

**Conclusion:**

This study demonstrated the reliability of current approaches that elevate the similarity of GO terms to the similarity of proteins. Suggestions for further improvements in functional similarity analysis are also provided.

## Background

Gene ontology (GO) [1] describes gene products based on their functions and is a structured and controlled vocabulary that has become quite popular among the known taxonomies. The root ontology (ALL) of GO consists of three independent terms: biological process (BP), molecular function (MF), and cellular component (CC). GO data provides a novel way to measure the functional relationship between gene products, which is the basis of most gene correlation studies [2]. Researchers interested in functionally related genes always hope to improve the accuracy of the results beyond the boundaries of currently available experimental data. Addition of knowledge data, for example, by computing the semantic similarity between genes may partially address this problem. Most semantic-based applications follow a three-step approach that includes semantic similarity calculations of paired GO terms, functional similarity calculations of all possible combinations of related GO terms, and further studies such as clustering analysis [3-6]. However, optimization of the methods for elevating the similarity of GO terms to the similarity of proteins is still required.

In semantic-based applications, it is necessary to compute the similarity among GO terms before investigating the similarity between gene products. Fortunately, the method for calculating two terms in the well-known semantic tree WordNet [7] has been well established. When GO emerged, these measures were widely used for determining the GO lexical instinct. In 2003, Lord *et al*. [8] found that sequence similarity was almost consistent with semantic similarity. Since then, several approaches have been developed that range from traditional methods in which the distance of two given nodes is calculated [4,5] to information-theoretic models in which the overall information of the tree structure is measured [3,6]. Similarity information derived from GO has also supported functional module studies [9]. There have been many reviews [10-12] in favour of information theory-based methods such as those proposed by Resnik [13] and Lin [14] since these methods are not sensitive to link densities, which are a key limitation of distance-dependent measurements. In this study, Resnik's method was chosen because it showed the best performance in most evaluations.

**Figure 1:**
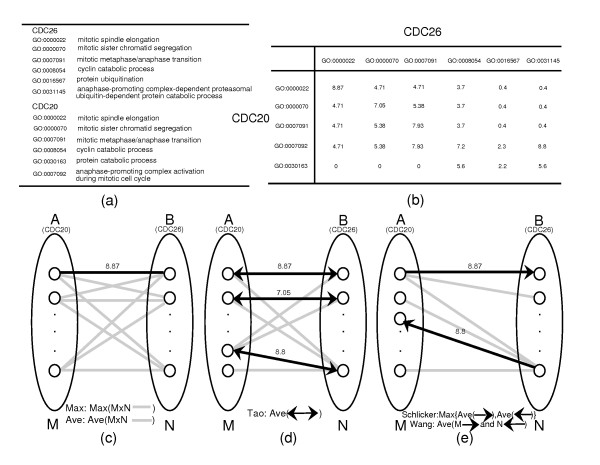
**Graphical illustration of functional similarity measures**. A summary of the current functional similarity approaches is provided in parallel to give an overview. (a) GO annotations of *CDC20 *and *CDC26 *in BP ontology. (b) Semantic similarity scores (from the Resnik method) between *CDC20 *and *CDC26 *annotations. *CDC20 *and *CDC26 *annotated with M (5) and N (6) GO terms (represented by small circles in c, d and e) form two groups A and B. The straight lines represent the semantic similarity scores between any two GO terms in groups A and B. A total number of 30 (5*6) scores are generated between *CDC20 *and *CDC26*. (c) The functional similarities are defined as the maximum values and average values over the 30 semantic scores for the Max and Ave methods respectively. The functional similarity score of *CDC20 *and *CDC26 *for Max is 8.87 (score between GO:0000022 and GO:0000022, straight black line), while for Ave, the value is 3.89. (d) The bidirectional arrows indicate the reciprocal best-score relations among GO terms. For example, GO:0031145 has the best score (8.8) from GO:0007092 among the other terms and vice versa, and the score is accepted. The best term for GO:0016567 is GO:0007092, while the best one for GO:0007092 is GO:0031145. The scores between these terms will not be counted. Tao took an average of all the accepted scores. The result from the Tao method is 4.5. (e) The maximum semantic score (best hit) is determined from each term in Group A to the terms in Group B (the forward arrows), and the process is repeated vice versa for the backward arrows. M (5 for *CDC20*) best hits from A to B and N (6 for *CDC26*) from B to A are collected. Schlicker defined the similarity of two genes as the maximum value of forward and backward average scores. By further combining the information from the MF and CC ontologies and normalizing the result into the range [0, 1], the Schlicker functional similarity score of *CDC20 *and *CDC26 *is determined to be 0.47. Wang defined the functional similarity between A and B as the average of M plus N (11 in the case of *CDC20 *and *CDC26*, 5 from each term of *CDC20 *to *CDC26 *plus 6 from each term of *CDC26 *to *CDC20*) directional best hits. The score is 7.3.

Biologists are more interested in the relationship of gene products than in the scores of GO terms. Various functional similarity approaches have been attempted. In this paper, these methods are referred to as 'Max' [3,9,15], 'Ave' [6,8,10], 'Tao' [16], 'Schlicker' [17,18] and 'Wang' [19] (see Fig. 1 for a brief illustration of these methods). Z. Lei *et al*. [20] have evaluated various functional similarity measures (2 of these – Max and Ave – were published) to predict protein subnuclear localization using a well curated database NPD [21]. Unfortunately, it was difficult to find a gold standard for assessing the functional relationship. So we merged the evaluation standards used earlier in semantic similarity research, i.e., we used both protein-protein interaction (PPI) [12] and gene expression datasets [11] of *S. cerevisiae*. The five above-mentioned methods were compared, and we hope that the results would provide a basis for further GO-based similarity studies.

## Results

### Assessment of functional similarity based on protein-protein interactions

The functional similarity methods introduced above and abbreviated as Max, Ave, Tao, Schlicker and Wang were tested by the receiver operating characteristics (ROC) analysis. ROC grades the performance of classifiers and rankers as a trade-off between specificity and sensitivity. The area under the ROC curve (AUC) is often taken as a measure of the prediction performance. An area of 0.5 represents random forecasts, while an area of 1 reflects perfect forecasts. A total of 6,459 *S. cerevisiae *protein interactions were retrieved from the Database of Interacting Proteins (DIP) [22] and filtered on the basis of the reliability of their GO annotation. We used 5,946 protein interactions (including 2,466 proteins) annotated in BP, 4,267 (1,945 proteins) in MF, 6,121 (2,534 proteins) in CC and 4,088 (1,850 proteins) in ALL as the positive datasets. The negative datasets containing the same number of protein pairs were randomly established based on the requirement of the ROC analysis (see Methods).

**Figure 2:**
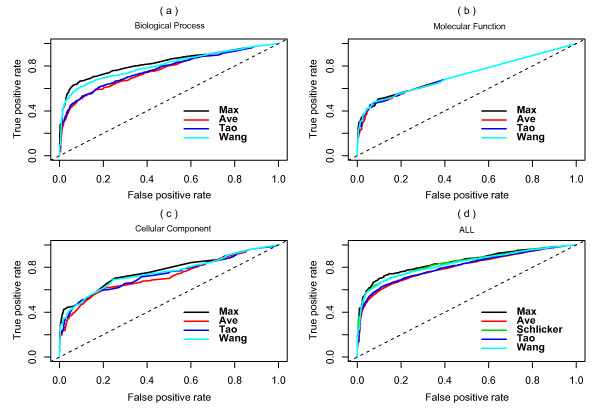
**ROC curves of PPI evaluations**. ROC evaluations of functional similarity measures based on the *S. cerevisiae *PPI dataset derived from DIP are shown. The evaluation was done in (a) biological process (BP), (b) molecular function (MF), (c) cellular component (CC), and (d) ALL (root ontology). Since the Schlicker method requires all three ontologies, it is only suitable in ALL.

Unexpectedly, the Max method consistently showed the best performance in spite of the fact that the performances of all measures were barely distinguishable in MF ontology (Fig. 2). The AUC values in Table 1 provide more details. The Max and Schlicker methods were adequate in BP ontology and were followed by the Wang, Ave and Tao methods. Since the tested functional similarity measures would give different results only when the genes were annotated by multiple GO terms (refer to Fig. 1), the number of genes annotated by a single GO term was investigated. In contrast to 34.6% in BP and 45.1% in CC, the number of genes annotated by single GO identifiers in MF was as high as 74% (Table 2). Interestingly, the gene numbers were distributed differently in each ontology. There were slight variations in the gene numbers in BP ontology among the single, double, triple and higher annotations. CC and MF were likely to assign less annotation terms to genes. This bias was clearer in MF than in CC. Most of these single annotations belonged to particular GO catalogues. For example, there were 43% MF single annotations in 'catalytic activity' and 27% in 'binding', 57.6% CC single annotations in 'organelle' and 28.1% in 'macromolecular complex', and 58% BP single annotations in 'metabolic process' and 23.3% in 'localization'. These results imply that genes involved in catalytic and binding activities were mostly of the 'one gene for one activity' type. Most genes localized in organelles and macromolecular complexes have unique locations in the cell. Some genes in metabolic and localization processes are unique to the particular process.

**Table 1:** Areas under ROC

Ontologies	Max.	Ave.	Tao	Wang	Schlicker
All (Root)	0.847	0.787	0.766	0.826	0.841
Biological Process	0.829	0.765	0.770	0.806	-
Molecular Function	0.722	0.715	0.717	0.718	-
Cellular Component	0.768	0.724	0.738	0.753	-

Areas under the curve (AUCs) are listed to provide detailed information on the PPI evaluation. Since the Schlicker method requires all three ontologies, it is only suitable in root ontology (ALL).

**Table 2:** The combined GO annotations of *S. cerevisiae*, *M. musculus *and *H. sapiens *genes in BP, MF and CC

Ontologies	4 annotations^a ^(%)	3 annotations (%)	2 annotations (%)	1 annotation (%)
*S. cerevisiae *BP	22.2	15.4	27.8	34.6
MF	1.4	5.3	19.3	74.0
CC	8.4	14.4	32.1	45.1
*M. musculus *BP	19.6	9.2	31.5	39.7
MF	0	0.7	41.8	57.5
CC	0	0.6	39.6	59.8
*H. sapiens *BP	17.5	12.5	24.9	45.1
MF	0	1	48.7	50.3
CC	0	0.7	42.3	57.0

Gene numbers (in percentage) annotated by various combinations of GO terms of BP, MF and CC are listed for the *S. cerevisiae*, *M. musculus*, and *H. sapiens *datasets.

^a^More and include 4 annotations

**Figure 3:**
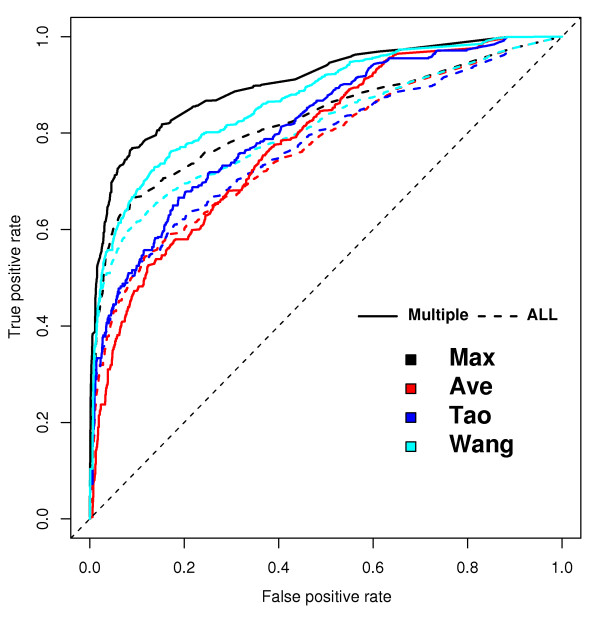
**Improvement of ROC curves after elimination of single annotations from the test dataset in the BP ontology**. The four methods (Max, Ave, Wang and Tao) are plotted in separate colours. The performances of the methods after eliminating single annotations are shown by the solid lines. Results based on the original dataset are shown by the dashed lines. The Schlicker method was not shown because it requires more ontology than BP.

To highlight the contribution of multiple-term annotations, we compared the ROC curves derived from BP and observed that all methods performed better when single-term annotations were abolished (Fig. 3). However, removal of single-term annotations led to less improvement in the case of the Ave and Tao methods but to approximately 9% improvement in the Max method. Additional experiments with MF and CC returned very similar results [see Additional file 1]. However, the AUC improvements were obtained at a cost. Approximately 60% of the protein interactions were not covered when single-term annotations were eliminated. Note that in the methods section, the number of protein interactions decreased from 5,946 to 2,414 in the test dataset that contained only multiple annotations.

The PPIs from the Munich Information Center for Protein Sequences (MIPS) Comprehensive Yeast Genome Database CYGD [23] (14,545 interactions and 5134 proteins, release date: 19 April 2007) were manually compiled from the literature and published large-scale experiments. 12741 interactions and 4343 proteins that have GO annotations were used in this additional evaluation. Therefore, we performed the same analysis for the yeast PPI data set from the CYGD database. The results were similar to those described above. For example, the AUC values of BP ontology were as follows: Max, 0.73; Ave, 0.67; Tao, 0.67 and Wang, 0.70. The details are shown in the supplementary file [see Additional file 2 and 3].

### Assessment of functional similarity based on microarray data

**Figure 4:**
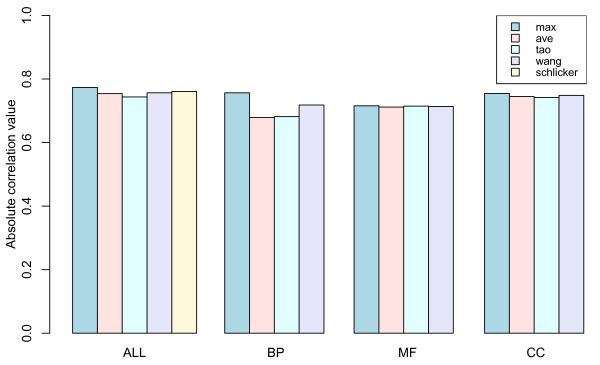
**Histogram of correlation coefficients**. The correlation coefficients between gene expression correlation and semantic similarity are compared among the tested methods. Since the Schlicker method requires all three ontologies, it is only suitable in root ontology (ALL).

Another indicator dataset of the gene function relationship was obtained from microarray data. In this study, Eisen's [24] dataset was used. Comparison of the correlation coefficients between gene expression correlation and semantic similarity are shown in Fig. 4. All high correlation values showed the feasibility of our evaluation approach, which has been reported earlier [11]. Similar to the results of the PPI test, the Max method exhibited the best performance, while the Ave and Tao methods showed the weakest correlation. The correlation coefficients of ALL indicated that the Max and Schlicker methods outperformed the other methods. The performance of all methods was similar for the MF and CC terms. The statistics of single annotations in these datasets showed 73.1% of genes in MF ontology (highest value), 39.8% in BP (lowest value) and 45.8% in CC.

**Figure 5:**
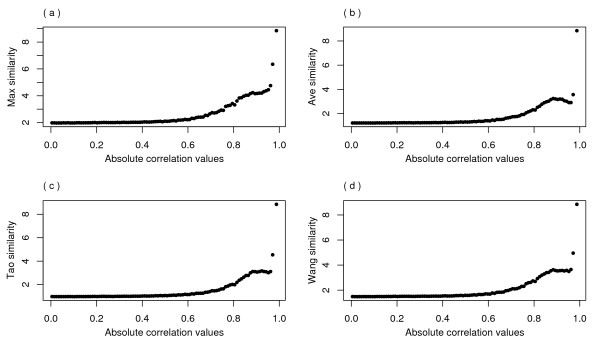
**Relationship of gene expression correlation and gene functional similarity in BP ontology**. This figure shows gene expression correlation (X axis) against gene functional similarity (Y axis) in BP ontology over 100 gene expression intervals of Eisen's dataset in *S. cerevisiae*. Various methods are compared: (a) Max, (b) Ave, (c) Tao and (d) Wang.

Test data in BP ontology were chosen to further analyse the relationship between the semantic similarity and correlation of gene expression. As depicted in Fig. 5, all functional similarity measures exhibited a trend in which higher expression correlation had stronger semantic similarities. The linearity was noticeable when the expression correlation value was above 0.6. While the other methods resulted in an uneven growth curve that was approximately 0.9 of the expression correlation value, the curve of the Max method tended towards a stable increasing trend.

## Discussion

In this study, we used the expression and PPI datasets of *S. cerevisiae *to evaluate five popular functional similarity algorithms. Applications of these datasets as statistical standards have been widely reported [4,6,25-28]. Two evaluation approaches were used because information on gene function obtained from various lab studies has shown that no single dataset would be ideal for testing a knowledge database. In this study, 1,226 proteins and only 36 protein pairs were found to overlap between the PPI and expression datasets. The majority of the proteins had different interactions in these two datasets. Note that in the case of expression data, only gene pairs that had an absolute correlation value exceeding 0.6 were regarded as expression-related genes because little linearity was detected at lower values, as shown in Fig. 5. Thus, 624 proteins and 4,052 interactions were uniquely represented by the PPI dataset, and 1,229 proteins and 69,024 correlations were unique to the expression dataset. The majority of unique genes, and consequently unique relationships, in these two datasets supported our assumptions. Although our results on the performance of functional similarity measures were quite promising, unexpected semantic similarities may have been obtained due to poorly annotated genes. This problem may be minimized in the near future as gene annotations are continuously refined. Inconsistencies in individual examples did exist between the PPI and expression datasets. For example, approximately 25% of the interacting proteins in *S. cerevisiae *had an unexpectedly low expression coefficient (below 0.1). A plausible reason for this is that 79 expression profiles in Eisen's data may not be sufficiently sensitive for detecting the interacting proteins; therefore, more expression data would be required. On the other hand, false positives are unavoidable in the PPI dataset. Another possibility is that these genes are simply not related at the expression level. Therefore, the use of multiple standards (PPI and expression data) to evaluate the performance of functional similarity approaches has the advantage of both coverage and reliability.

In all tests, the Max method consistently showed the best performance. Shared annotations or closely related GO terms, which lead to high semantic scores, was probably one of the reasons. There were 2,622 gene pairs (of 5,946) that had semantic scores of more than 6, and 2,278 (87% of 2,622) were contributed by shared GO terms in the multiple annotation dataset of BP ontology. To obtain biological details, two case studies are presented, one of which was supported by expression data and the other by PPI data. First, we considered two cell division control proteins CDC20 and CDC26 that have similar expression profiles. Although there is no PPI information on these two genes, their significant coexpression correlation value of 0.77 suggested functional relationships, which was also indicated by their shared GO annotations (refer to Fig. 1) and confirmed by other studies [29,30]. The functional similarity score calculated by the Max method was 8.9. *CDC20 *and *CDC26 *also have unique annotations such as 'anaphase-promoting complex activation during mitotic cell cycle' and 'protein ubiquitination', which led to some low semantic scores, and the Ave method was tuned to obtain a score of 1.9. Moreover, two interacting proteins SEC23 and BOS1 (described in the DIP dataset) have several annotations in which 'ER to Golgi vesicle-mediated transport' is their shared term. According to an earlier report [31], their PPI occurs during the process of 'ER to Golgi vesicle-mediated transport'. The semantic similarity deduced by the Max method was 9.5 whereas that obtained by the Ave method was 0.8.

In these examples, the average method that equilibrates all related semantic scores may compensate for some annotation mistakes but apparently leads to a much lower functional similarity score. The Wang and Tao methods, in which the best hits of each GO term subset are applied, enhanced the accuracy. However, the average of all best hits still led to a relatively low score. The Max and Schlicker methods, which gave the best scores, showed much better results. Although genes annotated with multiple terms may be associated in several ways, the most likely is through strong relationships, usually indicated by their shared terms or closely related terms. Other unique annotations or non-related annotations usually result in noise during the calculation of functional similarity. However, it is necessary to acknowledge that in the Max method, any annotation mistake may lead to false positive results. Note that in Fig. 2, the performance of the Ave method was relatively stable when the test dataset changed. Based on our preliminary findings and the conclusion that the sum of similarity scores of matched GO terms for two proteins shows best performance when applied to subnuclear localization prediction [20], use of a weighted average of all related semantic scores in favour of multiple shared terms may yield better results than any referred algorithms since there is a lower possibility of false annotations when multiple shared terms are used. In future studies, we will introduce an improved algorithm and some new software tools.

The functional similarity methods were tested in ALL, BP, MF and CC ontologies to evaluate their respective performances. As shown in Table 1, most methods consistently showed the best performance in ALL, better in BP and worst in MF. Note that the best performance in ALL resulted from our unique approach to test data collection. The ALL dataset contained the least number of proteins and protein interactions. For an inclusive ALL dataset that provides widespread protein coverage in BP, MF and CC (containing 2,764 proteins and 6,424 positive protein interactions), the performance will drop below that of the BP ontology (0.785 and 0.676 for the Max and Schlicker methods respectively). To trade-off coverage for performance, BP annotations would be the best choice, while the MF dataset would be the least informative among the *S. cerevisiae *datasets. New functional similarity algorithms need to consider different weights for the contributions of BP, MF and CC to obtain good performance and coverage.

In order to explain the worst performance or least informative character of the MF dataset of *S. cerevisiae*, the number of genes with single/multiple annotations were collated as shown in Table 2 and compared with those in BP and CC. It is very likely that the functional methods were not distinguishable in MF ontology because of the high proportion of single-term annotations, which were much fewer in BP. This raises the question of whether multiple annotations were responsible for the good performance of the BP dataset. Further analysis in BP ontology confirmed this possibility. Multiple-term annotations would lead to a more reliable functional similarity calculation. However, the AUC improvements were obtained at a cost. Approximately 60% of the protein interactions were not covered when single-term annotations were eliminated. In order to obtain high accuracy as well as extensive coverage, multiple-term and single-term annotations should be considered, but these should be treated differently. Table 2 shows an example of the distribution variations of single/multiple GO annotations in *S. cerevisiae*. Similar scenarios are observed with the human and mouse datasets; thus, if such functional similarity algorithms are extended to higher organisms and if multiple-term and single-term annotations are treated differently, as shown here, the results are expected to be quite promising.

Our evaluation of functional similarity approaches was based on *S. cerevisiae *datasets that have been continuously revised and improved. These datasets contain sufficient data that can be used to obtain accurate results. The results would contribute to the automated integration of prior and background knowledge in large-scale biological data mining. In particular, it provides good supporting information and suggestions for improving current and future applications of semantic similarity algorithms, such as functional similarity search tools [32], mRNA coexpression analysis, PPI prediction [27] and gene clustering.

## Conclusion

Five popular functional similarity methods were evaluated using PPI and expression datasets of *S. cerevisiae *to obtain sufficient gene coverage and reliable results. The tests were consistently in favour of the simple maximum method. The results suggested that functional similarity algorithms should introduce different weights for the BP, MF and CC terms and for multiple annotations. In particular, multiple and single annotations should be treated differently for greater reliability together with total coverage. Although these findings were based on the information obtained from the *S. cerevisiae *datasets, there is a good possibility of extending this study to higher organisms such as humans and mice. Functional similarity in favour of knowledge represented by GO will contribute more to gene function studies in the near future.

## Methods

### Data acquisition and data processing

The GO annotation files were downloaded from SGD released in October 2007 and contained 23,814 GO terms subdivided into 13,916 biological process (BP) terms, 7,879 molecular functions (MF) terms and 2,019 cellular component (CC) terms. These vocabularies possessed a spindle distribution along 16 levels of depth, where the 8th level contained most of the terms. Genes inferred from electronic annotation (IEA) were eliminated from further analysis due to the lack of reliability.

We retrieved 6,459 distinct PPIs (including 2,772 proteins) in *S. cerevisiae *from DIP (release date: 7 October 2007). Considering that genes annotated with terms from the top levels of the directed acyclic graph (DAG) structure of GO would create noise, only those terms starting from the 3rd level and below (3rd–16th levels) were retrieved, resulting in 5,946 protein pairs (including 2,466 proteins) from BP, 4,267 pairs (1,945 proteins) from MF, and 6,121 pairs (2,534 proteins) from CC ontology. These were used as positive datasets for ROC curve analysis. For ALL (root ontology), 4,088 protein pairs that designated 1,850 proteins were used. Note that the ALL dataset contained the least number of proteins because the genes should simultaneously have the 3rd to 16th levels of annotations in BP, MF and CC ontologies.

The expression dataset was taken from the study of Eisen *et al*. that contained 79 gene expression profiles of *S. cerevisia*e. Since most of the proteins in the Eisen dataset were well annotated in GO, 2,461 non-IEA annotated genes were obtained.

### ROC curve analysis

ROC grades the performance of classifiers and rankers as a trade-off between specificity and sensitivity. The positive datasets are described above. The negative datasets with the same number of protein pairs were generated by randomly choosing proteins from the non-positive genes located in the GO annotation files. To distinguish the reliability of multiple GO annotations and single GO annotations, positive and negative datasets of PPI containing 2,414 protein pairs annotated by multiple GO terms were built for BP ontology. The ROC and ROCR libraries in the R programming language were employed to calculate the AUCs and draw the graphs [33].

### Pearson correlation analysis

Millions of gene pairs were derived from the Eisen [24] dataset for further analysis of the correlation between gene expression and semantic similarity. For each gene pair, the semantic similarity scores and absolute values of expression correlation were calculated. The well-known Pearson correlation was used to calculate the expression correlation. Based on Sevilla's [11] study, we split the gene pairs into 100 groups with respect to the absolute expression correlation values and then calculated the average of the correlation values and similarity scores in each interval. Finally, the correlation coefficients between expression correlation and semantic similarity were computed. In addition, our study separately covered four aspects (ALL: considers all hierarchies of GO, BP: biological process, MF: molecular function and CC: cellular component). The barplot library in the R programming language was used to visualize the correlation coefficient of all procedures.

## Authors' contributions

TX, YZ and LD jointly designed the study, TX participated in the evaluation analysis, TX and YZ drafted the manuscript. All authors read and approved the final manuscript.

## Supplementary Material

Additional file 1 **Improvement of ROC curves after elimination of single annotations from the DIP dataset in the MF and CC ontologies**. The four methods (Max, Ave, Wang and Tao) are plotted in separate colours. The performances of the methods after eliminating single annotations are shown by the solid lines. Results based on the original dataset are shown by the dashed lines. The Schlicker method was not shown because it requires more ontologies than BP. Click here for file

Additional file 2 **ROC curves of PPI evaluations**. ROC evaluations of functional similarity measures based on the *S. cerevisiae *PPI dataset derived from CYGD are shown. The evaluation was done in (a) biological process (BP), (b) molecular function (MF), (c) cellular component (CC) and (d) ALL (root ontology). Since the Schlicker method requires all three ontologies, it is only suitable in ALL. Click here for file

Additional file 3 **Improvement of ROC curves after elimination of single annotations from the CYGD dataset in the BP, MF and CC ontologies**. The four methods (Max, Ave, Wang and Tao) are plotted in separate colours. The performances of the methods after eliminating single annotations are shown by the solid lines. Results based on the original dataset are shown by the dashed lines. The Schlicker method was not shown because it requires more ontologies than BP. Click here for file
