# Supplementary figures and images for: Evaluation of GO-based functional similarity measures using S. cerevisiae protein interaction and expression profile data
Source: BMC Bioinformatics. 2008 Nov 6;9:472. doi: 10.1186/1471-2105-9-472 (PMC2612010; doi:10.1186/1471-2105-9-472)

Molecular Function

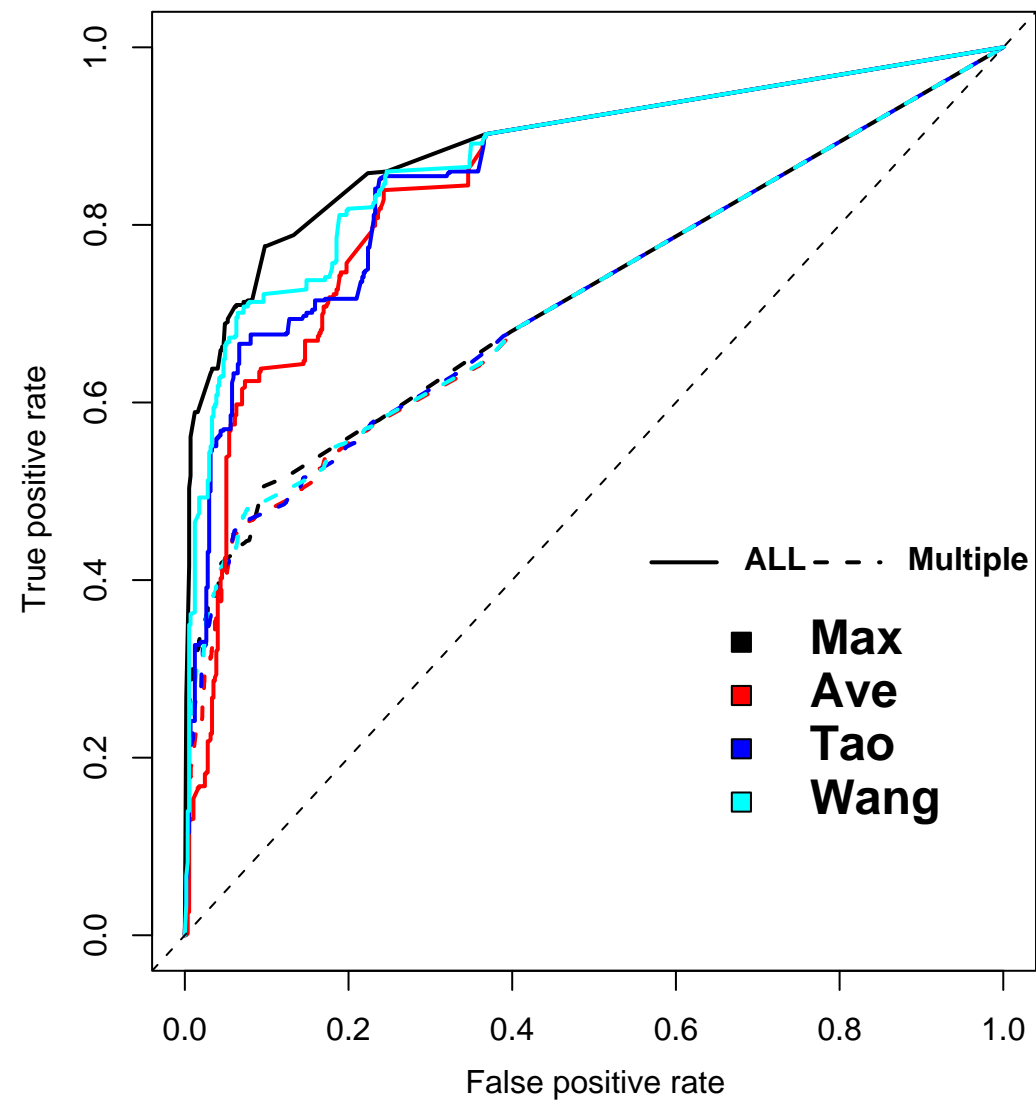

Cellular Component

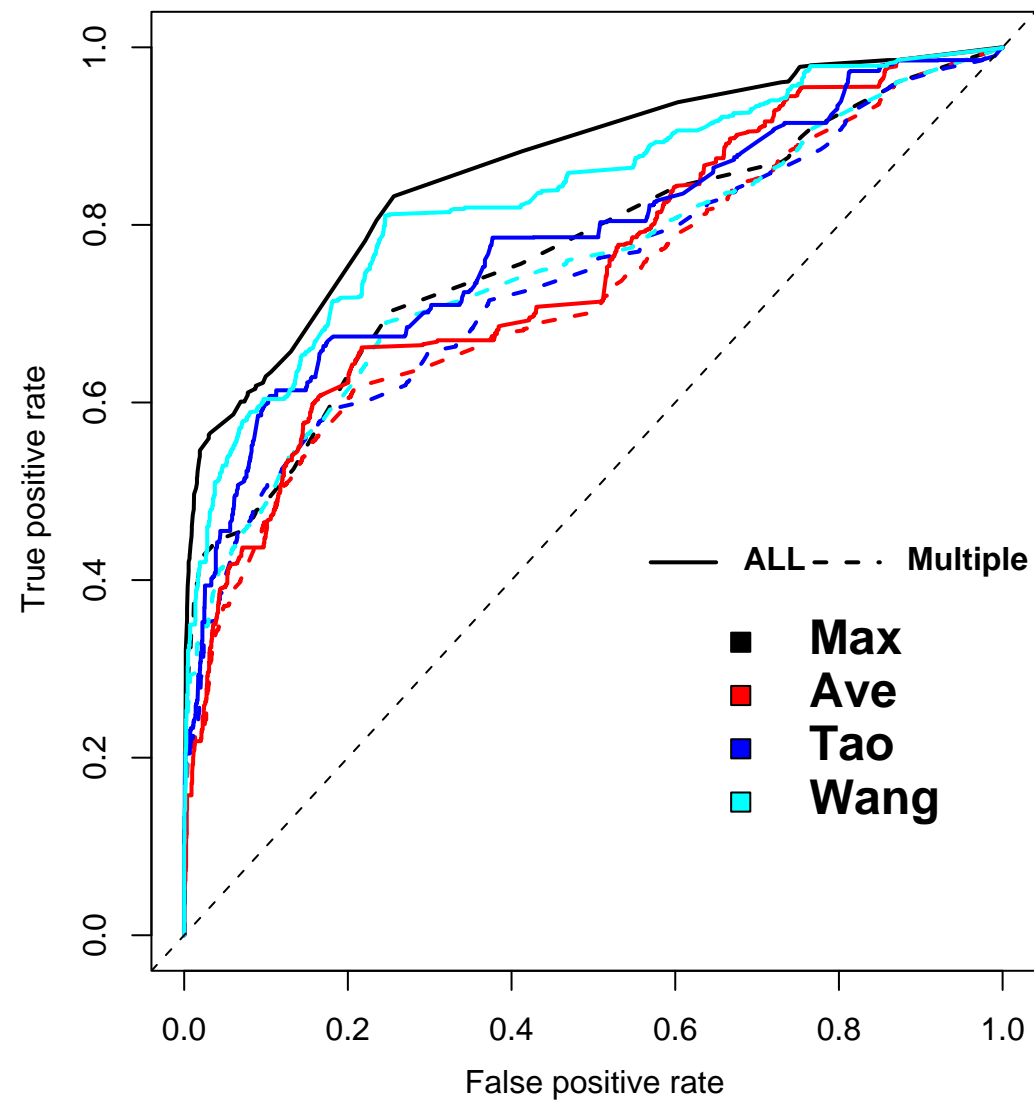

Supplement: Additional file 1 — Improvement of ROC curves after elimination of single annotations from the DIP dataset in the MF and CC ontologies. The four methods (Max, Ave, Wang and Tao) are plotted in separate colours. The performances of the methods after eliminating single annotations are shown by the solid lines. Results based on the original dataset are shown by the dashed lines. The Schlicker method was not shown because it requires more ontologies than BP. [file 1471-2105-9-472-S1.pdf]

(a)  
Biological Process

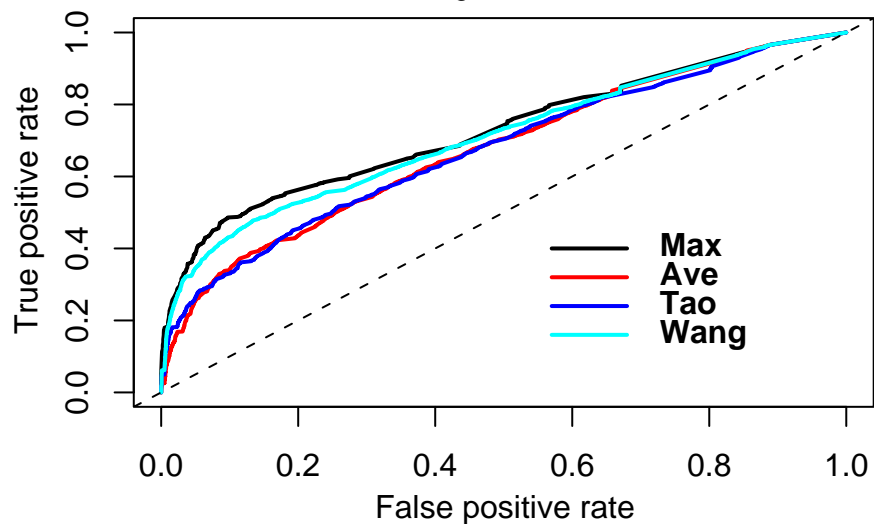

(b)  
Molecular Function

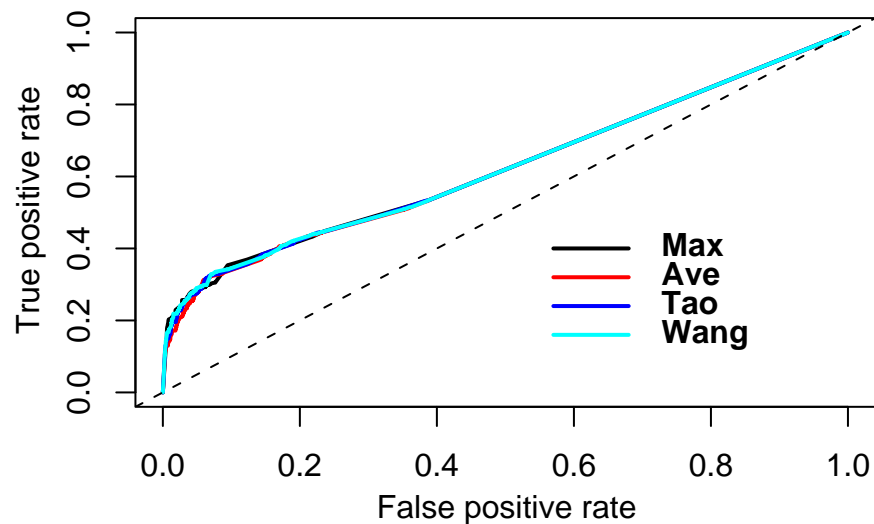

(c)  
Cellular Component

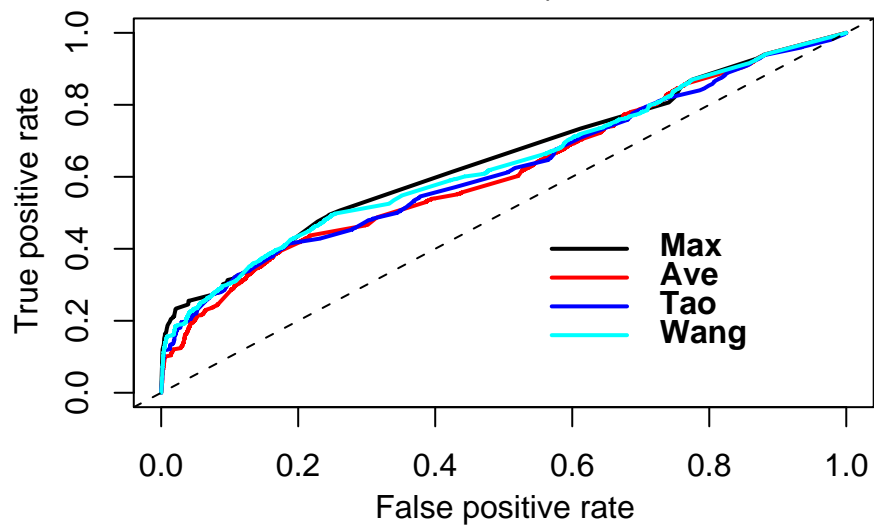

(d)  
ALL

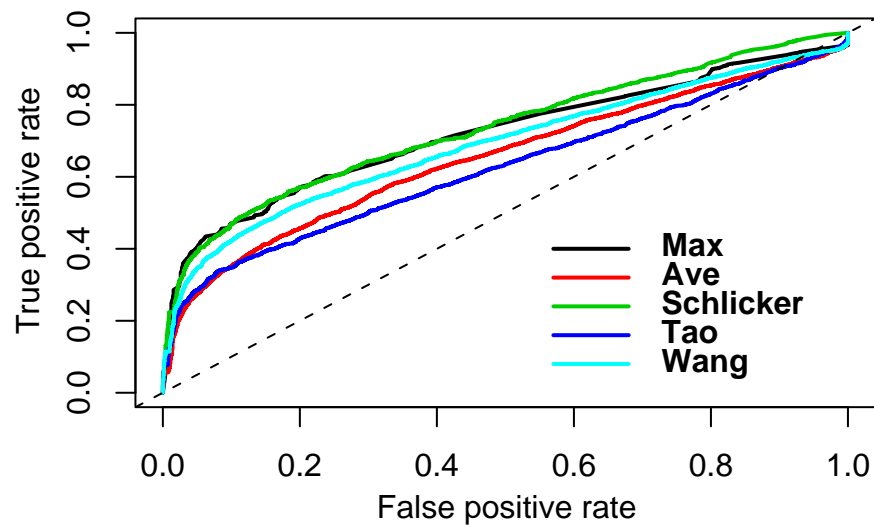

Supplement: Additional file 2 — ROC curves of PPI evaluations. ROC evaluations of functional similarity measures based on the S. cerevisiae PPI dataset derived from CYGD are shown. The evaluation was done in (a) biological process (BP), (b) molecular function (MF), (c) cellular component (CC) and (d) ALL (root ontology). Since the Schlicker method requires all three ontologies, it is only suitable in ALL. [file 1471-2105-9-472-S2.pdf]

Biological Process

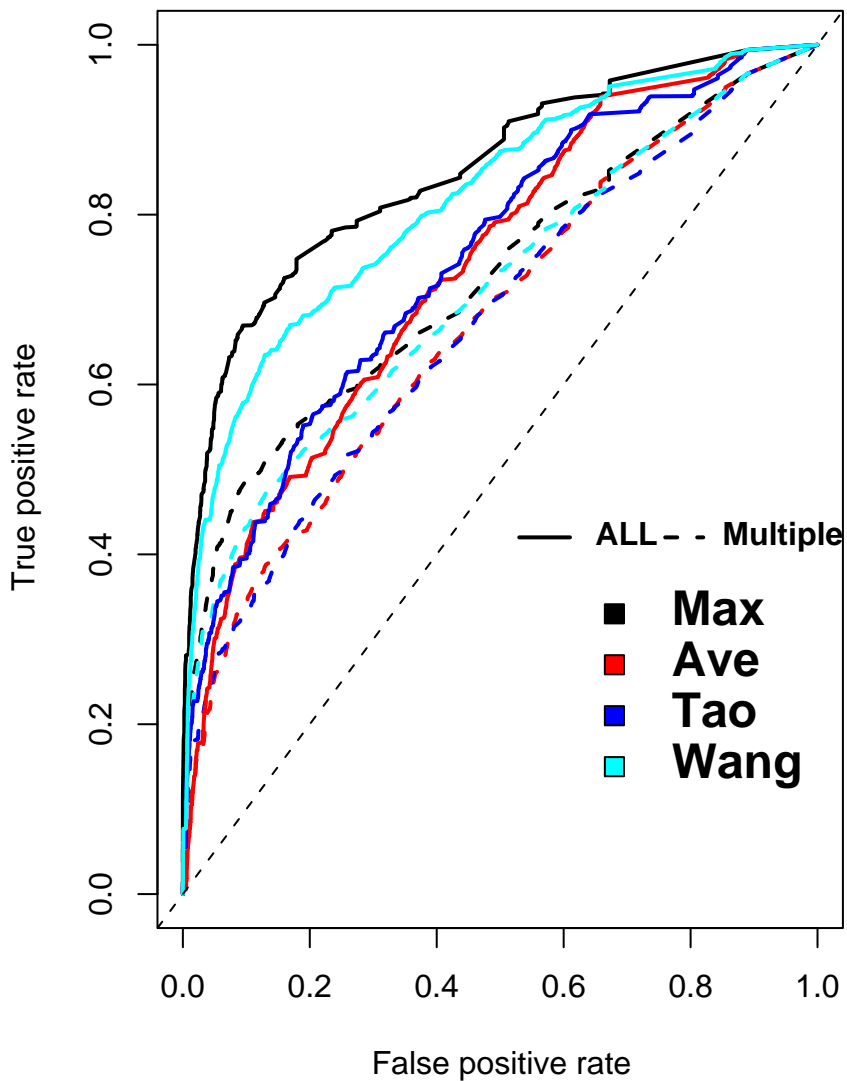

Molecular Function

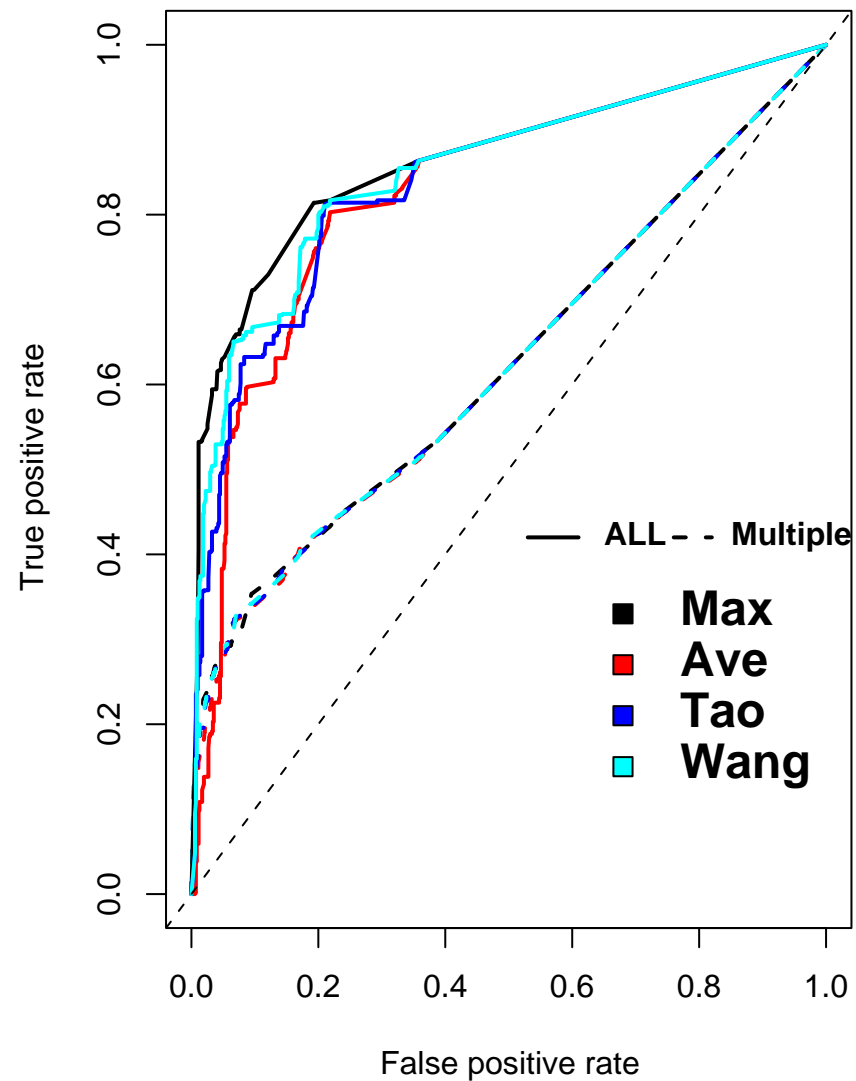

Cellular Component

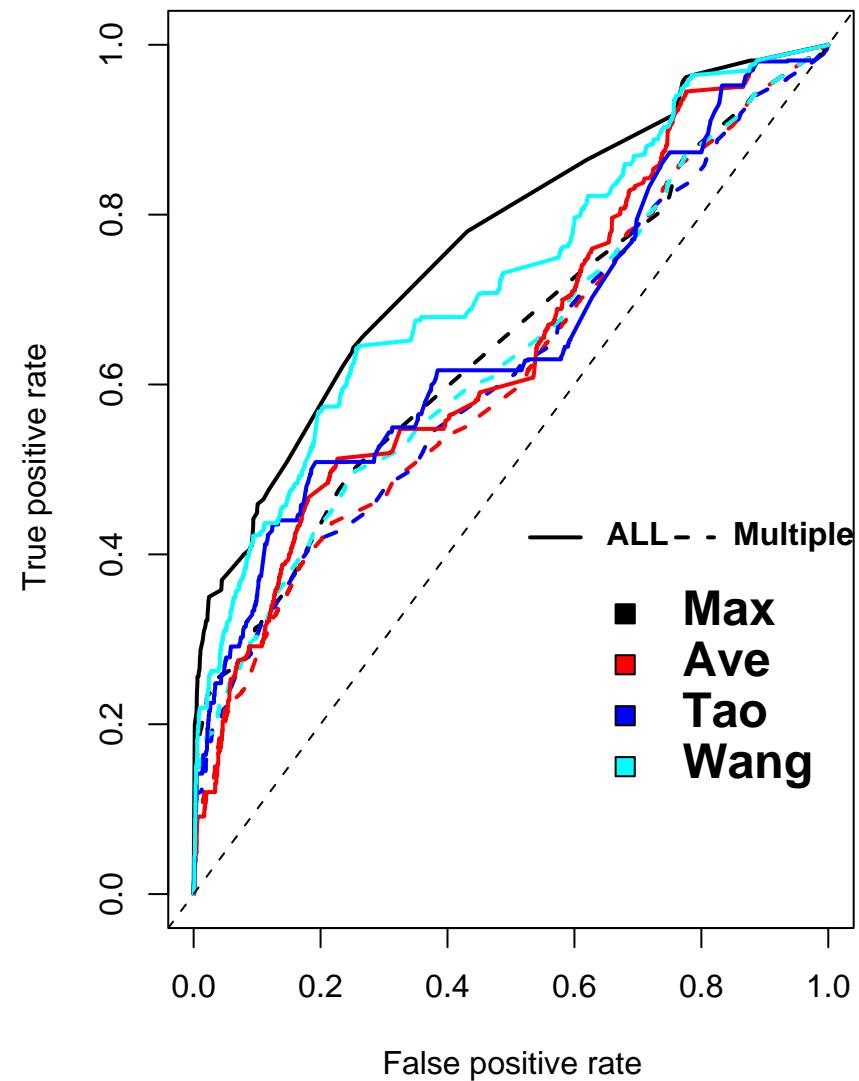

Supplement: Additional file 3 — Improvement of ROC curves after elimination of single annotations from the CYGD dataset in the BP, MF and CC ontologies. The four methods (Max, Ave, Wang and Tao) are plotted in separate colours. The performances of the methods after eliminating single annotations are shown by the solid lines. Results based on the original dataset are shown by the dashed lines. The Schlicker method was not shown because it requires more ontologies than BP. [file 1471-2105-9-472-S3.pdf]
